# A novel minimally invasive stereotaxic technique to target inner ear neurons in the mouse

**DOI:** 10.3389/fneur.2025.1696492

**Published:** 2026-01-05

**Authors:** Reef K. Al-Asad, Ngoc-Nhi C. Luu, Albert S. Edge, Judith S. Kempfle

**Affiliations:** 1 The Eaton-Peabody Laboratories, The Massachusetts Eye and Ear Department of Otolaryngology—Head and Neck Surgery, Boston, MA, United States; 2 University of Massachusetts Chan Medical School, Worcester, MA, United States; 3 Harvard Medical School Department of Otolaryngology—Head and Neck Surgery, Boston, MA, United States; 4 Department of Otolaryngology, UMass Memorial Medical Center, Worcester, MA, United States

**Keywords:** auditory neuropathy, sensorineural hearing loss, spiral ganglion neurons, stereotaxic surgery, hearing regeneration

## Abstract

Sensorineural hearing loss (SNHL), a leading cause of disability worldwide, arises from damage to hair cells (HCs) or spiral ganglion neurons (SGNs) within the cochlea. Among its etiologies, auditory neuropathy (AN) is characterized by disrupted signal transmission due to SGN damage. Traditional interventions, such as hearing aids and cochlear implants, provide limited benefit in cases of AN, where neuronal damage impairs signal transduction to the brain. Emerging regenerative therapies, including cell replacement and gene delivery, hold potential to restore SGN function, but their application is limited by challenges in delivering therapeutic agents to cochlear targets. In this study, we developed a novel stereotaxic approach for minimally invasive, precise delivery of therapeutic agents to murine SGNs. Utilizing pre-determined coordinates, we successfully accessed the cochlea and SGNs. Immunohistochemistry confirmed accurate delivery and integration of therapeutic agents. Functional hearing assessments showed that the approach preserved HC function and demonstrated minimal adverse effects. This technique offers a scalable platform for advancing cell and gene therapies aimed at restoring auditory function in AN and other forms of SNHL.

## 1 Introduction

Hearing loss affects over 430 million people globally and significantly impacts quality of life by reducing communication ability and increasing the risk of cognitive decline, depression, and social isolation (1). Sensorineural hearing loss (SNHL), the most prevalent form, arises from irreversible damage to the inner ear’s sensory or neural components (2). Within the cochlea, hair cells (HC) transduce sound vibrations into electrical signals that are relayed to the brain by spiral ganglion neurons (SGN) (3). Disruption of this pathway, whether due to HC loss, SGN damage, or synaptic dysfunction, results in varying degrees of hearing impairment.

Auditory neuropathy (AN) exists under the umbrella of auditory neuropathy spectrum disorders (ANSD), a subset of SNHL characterized by intact distortion product otoacoustic emissions (DPOAEs) but severely impaired auditory brainstem responses (ABR) (4–7). This condition can arise from primary SGN damage or auditory synaptopathy, where synaptic connections between HCs and SGNs are lost (8). Current treatments for hearing loss, such as cochlear implants or hearing aids, require intact SGNs to transmit sound signals effectively (9). Hearing aids have been provided to patients with milder forms of AN, albeit with limited to poor efficacy. Early cochlear implantation is indicated in patients with ANSD, particularly children (10). However, while being reported to provide some benefit, its efficacy in speech recognition is limited and variable (9, 10). Patients with lesions in the spiral ganglion have been found to have worse post-implantation speech recognition outcomes as compared to patients with HC lesions (11). Therefore, current treatment options for patients with AN have limited efficacy.

Recent advances in regenerative medicine offer alternative approaches to address AN. Previous studies have successfully differentiated embryonic stem cells (ESC) or induced pluripotent stem cells toward an auditory neuronal phenotype and transplanted them at different levels of differentiation into the murine cochlea to replace the damaged SGNs with varying degrees of graft integration (12–17). There has been significant study of the delivery and expression of neurotrophic factors and pro-neural genes in both protection of synapses and auditory nerve from damage, and auditory nerve regeneration. Additionally, gene therapy using viral vectors has been utilized in several studies to deliver neurotropic factors or pro-neural genes into the inner ear. Several studies have focused on the use of viral vectors carrying brain-derived neurotrophic factor (BDNF) or neurotrophin-3 (NT-3), which have demonstrated the ability to promote synaptic ribbon and SGN survival and regeneration (18–22). Direct delivery of neurotrophic small molecules has shown similar effects (23, 24).

Endogenous approaches relied on remaining cells to be stimulated with small molecules for signaling pathway manipulation or overexpression of proneural genes for regeneration (25, 26), and various studies have shown the potential of glial cells in the spiral ganglion to harbor progenitor qualities that allow reprogramming (27–30). Ultimately, the cochlea’s delicate and encapsulated anatomy poses significant barriers to effective delivery of any of these therapies.

Traditional surgical approaches for accessing the cochlea, particularly in small animal models, risk damaging the inner ear’s sensitive structures due to its minute size and complex geometry (31). This is further complicated when trying to access the modiolus, the central neural compartment of the inner ear encased in a bony canal. Previous studies that have attempted to deliver their experimental therapy, be it stem cells, viral vectors or small molecules, to the neural component of the inner ear have used round window membrane injections, semicircular canal injections, and cochleostomies (14, 31–33). Current methods for drug delivery or cell transplantation in murine models often pose substantial risks to hearing and surrounding structures, such as the disruption of cochlear fluid homeostasis or mechanical damage to delicate sensory cells. Such complications frequently outweigh the potential therapeutic benefits. As such, there is a critical need for minimally invasive techniques that allow precise delivery of therapeutic agents to cochlear targets.

Stereotaxic surgical techniques are widely regarded as essential tools for precise targeting within the central nervous system (CNS) of rodent and non-human primate models, owing to their minimally invasive nature and high reproducibility (34, 35). Clinically, such methods have been adapted and continuously innovated for a wide range of diagnostic and therapeutic interventions, allowing for more precise procedures with decreased morbidity. Among many applications, they have been utilized for brain biopsies, intracranial radiosurgery of tumors, and other brain pathologies, such as deep brain stimulator implantation for movement disorders (36–40). In the experimental setting, stereotaxic surgery is irreplaceable for accurate targeting of intracranial structures and delivery of materials in both non-human primates and rodents. Delivery requires a three-dimensional frame with fixed spatial coordinates, which enables accurate probe or cannula insertion through small burr holes, and provides a precision that minimizes human error, enhances reproducibility and allows for minimally invasive access (41).

Stereotaxic methods are well-established for CNS studies in animal models; however, their application to the inner ear has not been explored (42). This gap can be attributed to two significant challenges: the hearing portion of the inner ear, the cochlea, is encapsulated by dense bone, and it is situated within the lateral skull base, outside the standard boundaries of CNS-focused stereotaxic atlases, such as the Paxinos and Franklin Mouse Brain Atlas (43, 44). Despite this, considering the increased risk of cochlear damage caused by current methods of drug delivery or cell transplantation, stereotaxic access to the cochlea offers a promising avenue for advancing therapeutic strategies targeting the inner ear. The precise localization with stereotaxic techniques enables targeted access and controlled delivery of pharmacological agents, viral vectors or cells, to the auditory nerve as it exits the cochlea. By extending the reach of stereotaxic technology, which has proven its utility in CNS research and treatment paradigms, to the inner ear for the first time, this novel strategy may pave the way for translatable interventions for SNHL from AN.

This manuscript describes a precise, reproducible, minimally invasive surgical technique to deliver therapeutics that target the neuronal component of the cochlea in mice using stereotaxic apparatus.

## 2 Materials and equipment

### 2.1 Animals

6–8-week-old CBA/CaJ mice (The Jackson Laboratory, RRID: IMSR_JAX:000654).

### 2.2 Equipment

#3C Forceps (Dumont).

0.033 inch microdrill bit (Kyocera)

7–0 Prolene suture (Ethicon)

30-gauge needle

36-gauge needle

200S Readout System—Acu-rite

Confocal microscope (Leica Microsystems)

Cryostat (Leica Microsystems)

Drill arm attachment (Kopf Instruments)

Microinjector pump (World Precision Instruments)

Neurosyringe (Hamilton Company)

Stereotaxic apparatus (Kopf Instruments)

Syringe holder attachment (Kopf Instruments).

### 2.3 Reagents

Antibody solution: 0.1% Triton and 10% normal goat serum or normal donkey serum in PBS

Blocking solution: 0.3% Triton and 15% normal goat serum or normal donkey serum in PBS

Buprenorphine, 0.05 mg/kg in 0.9% NaCl

Citrate buffer (1% in distilled water)

Coverslips (ThermoFisher 24×50 #1)

DAPI (BD biosciences, 564,907)

Ethylene-diamine-tetra-acetic acid (EDTA, 0.12 M)

Ketamine, 100 mg/kg in 0.9% NaCl

Meloxicam, 2 mg/kg in 0.9% NaCl

Mouse anti Beta Tubulin III (TUJ1), 1:300 (Biolegend 801,202, RRID: AB_2313773)

Optimal cutting temperature (O. C. T.) compound (Tissue-Tek)

Ouabain, 1 mM in distilled water (Sigma)

Paraformaldehyde, 4% in PBS

Phosphate buffered saline (pH = 7.4)

Rabbit anti GFP, 1:250 (abcam, ab13970)

Secondary antibodies (Alexa Fluor, Invitrogen)

Sucrose, 5, 30% in distilled water

Triton, 0.3% 100X triton in PBS

Xylazine, 20 mg/kg in 0.9% NaCl.

## 3 Methods

### 3.1 Study design

This study aimed to develop and validate a minimally invasive stereotaxic method for delivering therapeutic agents to SGNs in mice. Targeting accuracy and integration of transplanted cells were evaluated and optimized to ensure the validity of this protocol. Ethical approval was obtained from the Institutional Animal Care and Use Committee (IACUC) at Massachusetts Eye and Ear Infirmary.

### 3.2 Animal model

Six- to eight-week-old male CBA/CaJ mice (The Jackson Laboratory) were used for all experiments due to their well-characterized auditory physiology and suitability for cochlear research. Animals were housed under standard laboratory conditions with ad libitum access to food and water.

### 3.3 Induction of auditory neuropathy

**Figure 1:**
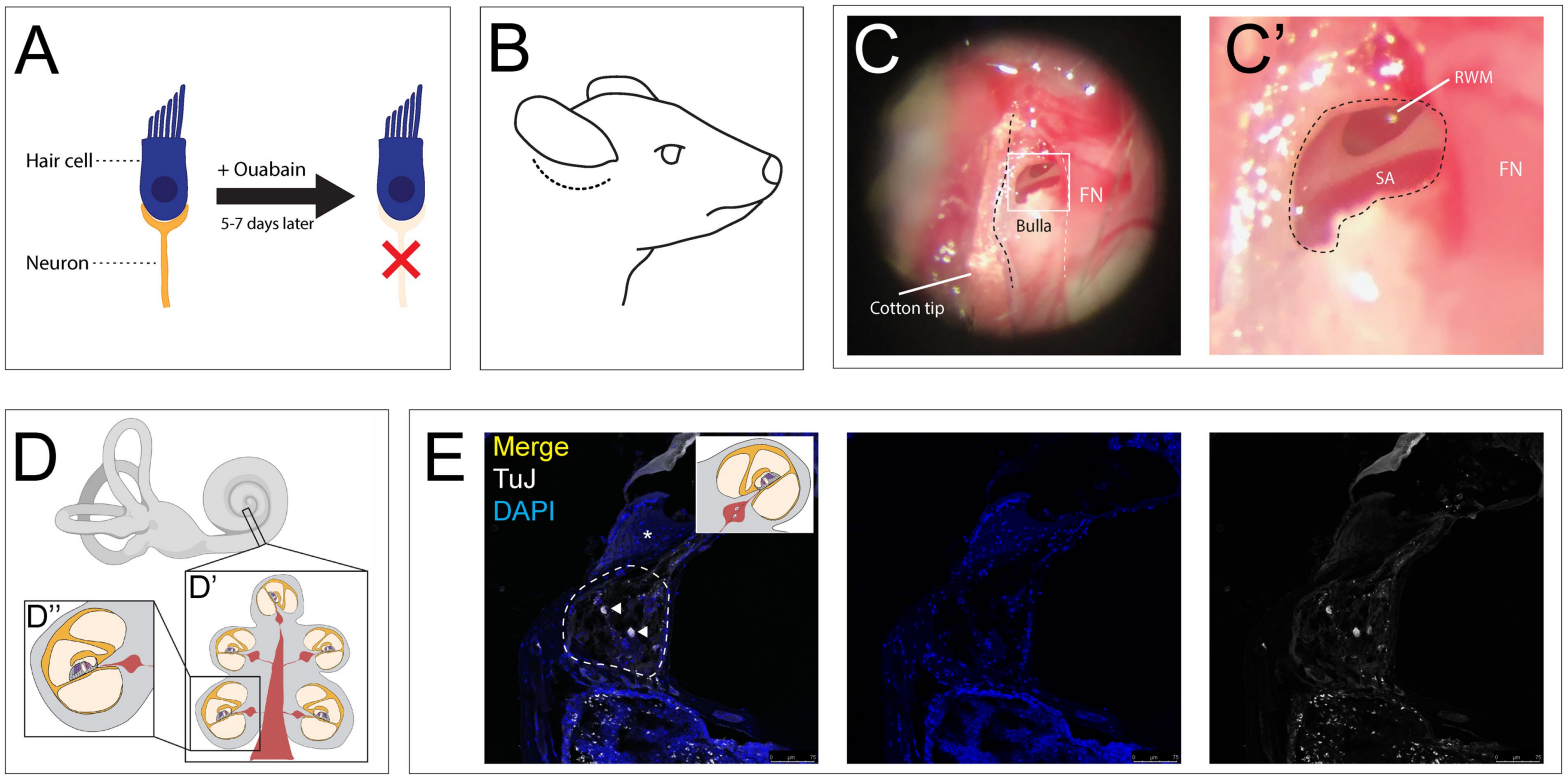
Ouabain application to the round window membrane damages auditory neurons while preserving hair cells. **(A)** Ouabain acts to selectively damage auditory neurons without affecting hair cells. **(B)** Illustrated lateral view of mouse head with post auricular incision (dotted line). **(C)** Low magnification view of the bulla with an opening to expose the round window membrane, with facial nerve (FN, white dotted line) serving as a landmark for weak spot in the bulla. **(C″)** High magnification image of RWM superior to the stapedial artery (SA). FN seen adjacent to hole made in bulla. **(D)** Illustration of the inner ear, including the cochlea and vestibular apparatus. **(D′)** Mid-modiolar cross section through the cochlea, showing the turns of the cochlea containing the organ of Corti, Rosenthal’s canals containing SGN cell bodies, with central projections making up the modiolus. **(D″)** Illustration of single half-turn of the cochlea and Rosenthal’s canal. **(E)** Coronal section of cochlea treated with Ouabain, showing Rosenthal’s canal (dotted line) and a cochlear half turn with Organ of Corti (asterisk), stained with TUJ1 (grey) and DAPI (blue). Two remaining neurons can be seen after treatment (white arrowheads). Created in BioRender. Kempfle, J. (2025) https://BioRender.com/kvin64n.

Auditory neuropathy was induced by applying ouabain, a sodium-potassium ATPase inhibitor, to the RWM as previously described (Figure 1A) (45). 6–8-week-old CBA/CaJ mice were anesthetized with ketamine (100 mg/kg, intraperitoneal (i.p.)) and Xylazine (20 mg/kg, i.p.), and received an additional half dose every 30 min. A postauricular incision was made behind the right pinna (Figure 1B). The trapezius and sternocleidomastoid muscles were retracted to reveal the facial nerve. The main trunk of the nerve was followed to expose the bulla, the bony covering of the middle ear. A 30-gauge needle was used to create a small opening in the superior aspect of the bulla, which was then widened with a #3C forceps (Dumont) to visualize the round window niche within the middle ear (Figures 1C, C″). Care was taken to keep the RWM intact so as not to violate the inner ear fluid space. Ouabain (1–2 μL, 1 mM in distilled water (Sigma)) was slowly applied to the RWM using a 36-gauge needle on a 10 μL Hamilton syringe (Hamilton Company) and replaced every 10 min for 1 h (46). The incision was then closed using 7–0 Prolene sutures (Ethicon). The mice were placed on a heating pad to recover. Buprenorphine (0.05 mg/kg, q12h, subcutaneous, SQ) and Meloxicam (2 mg/kg, q24h, SQ) were given for postoperative analgesia.

### 3.4 Auditory testing: auditory brainstem response and distortion product otoacoustic emissions

ABR testing provided a robust, quantitative measure of SGN function and the overall integrity of the auditory pathway. Additionally, to confirm selective neuronal damage without impacting HCs, we complemented ABR testing with DPOAEs to assess outer hair cell (OHC) function. Mice were anesthetized as described above and placed in a soundproof chamber. Three subdermal electrodes were inserted to record the auditory neuron response: The grounding electrode was placed at the tail, the active measuring electrode was fixed at the vertex, and the reference electrode was placed inferior and anterior to the pinna. A probe containing a speaker was inserted into the external auditory canal for emission of a “click” auditory stimulus. The ABR was recorded at seven frequencies (5.66 kHz, 8.00 kHz, 11.33 kHz, 16.00 kHz, 22.65 kHz, 32.00 kHz, and 45.24 kHz). Each frequency was presented at 5 dB sound pressure level (SPL) increments from 20 to 100 dB SPL. The ABR threshold was defined as the lowest SPL at which a recognizable waveform in the ABR was identified. Abnormal ABRs were defined as an increase in threshold of greater than 30 dB SPL. At the same time, DPOAEs were measured to confirm OHC function. A probe containing a speaker and microphone was carefully placed into the EAC. Two pure tone frequencies were presented at identical SPLs as above to elicit an otoacoustic emission from the OHC, which was recorded by the microphone (47). Abnormal DPOAEs were defined by an increase in threshold (46).

ABRs were recorded in anesthetized mice both before and 5–7 days after ouabain application. Successful induction of AN was indicated by significant elevations in ABR thresholds to levels exceeding 60 dB SPL, consistent with severe SNHL, and normal DPOAEs. Mice exhibiting abnormal DPOAEs were excluded from further experiments. Hearing assessments were repeated at 2 weeks, 1 month, and 3 months post-intervention to evaluate the long-term efficacy of therapeutic delivery.

### 3.5 Stereotaxic surgery

One week following ouabain-induced SGN damage, stereotaxic injections were performed on the right (ipsilateral) auditory nerve (Figures 1D, D’, D″, E). The contralateral ear served as an internal control, and additional sham-injected mice were included as procedural controls.

#### 3.5.1 Stereotaxic setup

**Figure 2:**
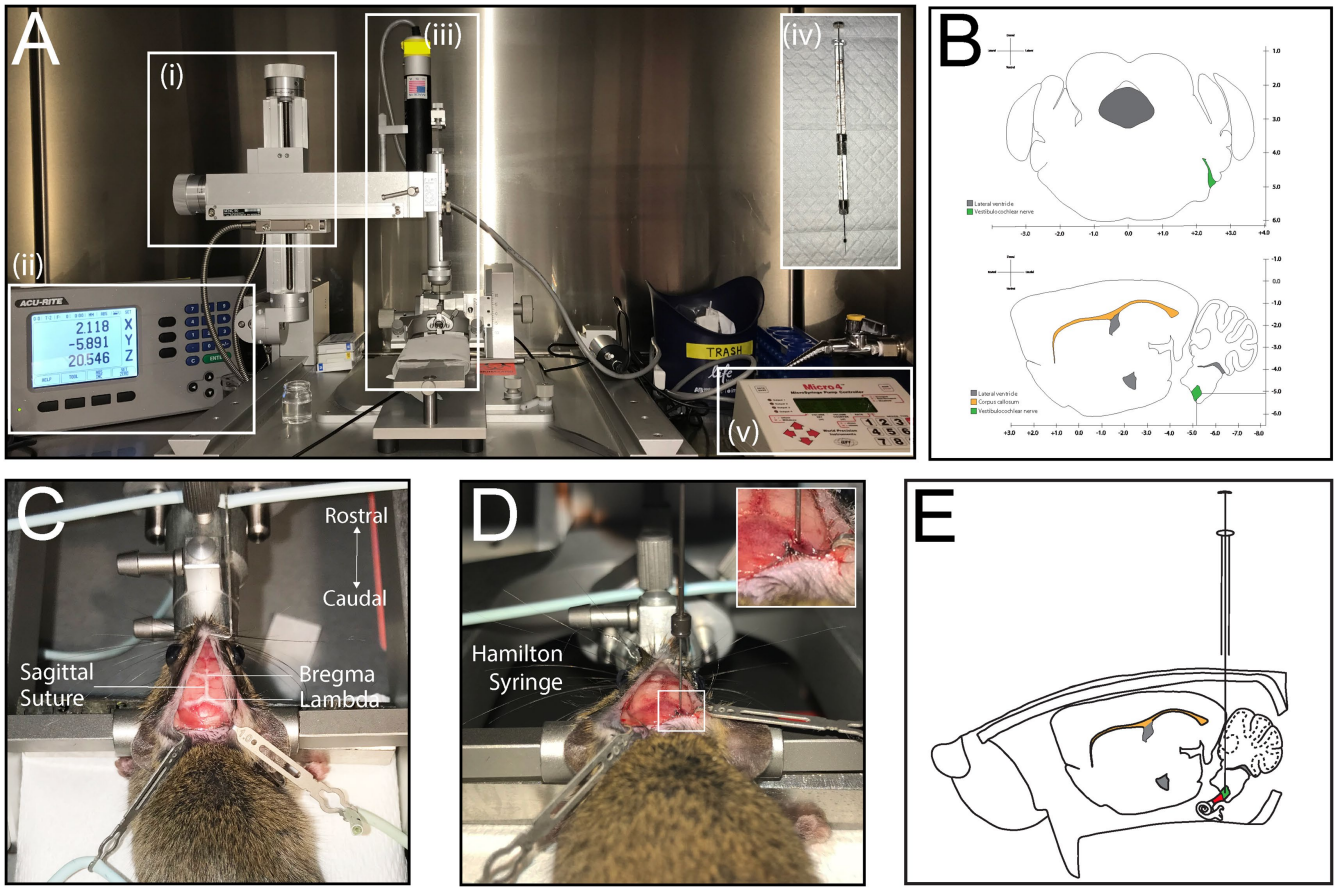
Stereotaxic injection to determine coordinates of the auditory nerve. **(A)** Set up of stereotactic apparatus prior to injection; coordinate readout system (i), stereotactic frame and arm attachment (ii), syringe holder and Hamilton neurosyringe (iii), Hamilton neurosyringe used for cell or virus injection (iv), Micropump (v). **(B)** Simplified illustration of coronal and sagittal sections of the mouse brain with coordinates of the auditory nerve as it exits the brain. **(C)** Dorsal view of mouse placed in stereotactic frame, with exposed skull showing the bregma, lambda and sagittal suture line. **(D)** Injection with Hamilton syringe through burr hole. **(E)** Illustration of injection into the mouse, sagittal view.

A stereotaxic apparatus (Kopf Instruments) was used, which consists of an X-Y-Z Cartesian coordinate frame with an attachment arm for a drill and/or injector. The arm allowed for movement along 3 axes: mediolateral (ML), dorsoventral (DV) and anteroposterior (AP) [Figures 2A(i)]. The arm was paired with a coordinate readout system (200S Readout System, Acu-rite) to track the X-Y-Z coordinates [Figures 2A(ii)]. The coordinates were selected based on the Paxinos and Franklin’s Mouse Brain Atlas, which contained coronal brain sections of 6-week-old C57/Bl6 mice and showed the exact coordinates of the auditory nerve trunk exiting the brain stem (Figures 2B,E). The target location, measured from the bregma, was AP = −5.11 mm, ML = +2.4 mm, and DV = 5.19 mm ± 0.1 mm. At the exact ML and AP coordinates, a bur hole craniotomy was created with a microdrill mounted to the drill arm attachment [Kopf Instruments, Figures 2A(iii)] and a 0.033-inch micro drill bit (Kyocera) (Figures 2A,C). The injection utilized a syringe holder attachment [Kopf Instruments, Figures 2A(iii)], with a 10 μL Hamilton neurosyringe (Hamilton Company) fitted with a 33-gauge needle for cell injection [Figures 2A(iv)]. A microinjector pump (World Precision Instruments) was connected to the syringe holder and allowed for a steady injection rate of 200 nL/min [Figures 2A(v)].

#### 3.5.2 Stereotaxic procedure

The mice (*n* = 21) were anesthetized and positioned in the stereotaxic frame with an adjustable incisor bar and ear bars, ensuring horizontal alignment of the skull. The surgical site was prepared by retracting scalp tissue and exposing bony landmarks, including the bregma and interosseous sutures, which were marked for coordinate reference (Figure 2C). A microdrill equipped with a 0.033-inch drill bit (Kyocera) was used to create a small bur hole through the skull, terminating at the dura mater. Any venous bleeding encountered, typically from the sigmoid sinus, was controlled using absorbable gelatin sponge (e.g., Gelfoam). The drill was then replaced with a Hamilton neurosyringe mounted on the stereotaxic arm, ensuring precise alignment with the pre-determined coordinates (Figures 2D,E). Cells were injected at a controlled rate of 200 nL/min using a microinjector. To prevent backflow or displacement of the injected solution, the needle was left in place for 5 min before being retracted slowly. The incision was closed with single-layer interrupted sutures (7–0 Prolene, Ethicon), and mice were monitored during recovery. The duration of this procedure required up 1 h per mouse; this time did not include set up of stereotaxic apparatus, which took about 15 min. To optimize efficiency, the authors of this study performed multiple procedures on the same day.

#### 3.5.3 Delivery of progenitor cells into the auditory nerve trunk

Tau-EGFP+ neural progenitor cells were generated from mouse embryonic stem cells (mESCs) and differentiated into neuronal precursors using previously established stepwise protocols (15, 48, 49). On the day of transplantation, cells were dissociated into single-cell suspensions and resuspended in cold culture medium. To maximize viability and prevent clumping, the cell solution was kept on ice for no more than 2 h prior to injection. The cells were counted after dissociation to ensure a consistent number of cells (~100,000).

To ensure that our method can accurately transplant cells into the correct location in the auditory nerve, we transplanted Tau-EGF mESC containing green fluorescent protein tag. Cells were delivered using a 10 μL Hamilton neurosyringe fitted with a 33-gauge needle, for a total injection volume of 3–5 microliters (μL) per mouse.

#### 3.5.4 Tissue processing and immunohistochemistry

Tissue was harvested for analysis after post-operative hearing assessments at 2 weeks, 1 month or 3 months.

Mice were anesthetized with ketamine [100 mg/kg, intraperitoneal (i.p.)] and Xylazine (20 mg/kg), i.p. Once the mouse no longer has reflex responses, it was euthanized by cardiac perfusion with 4% paraformaldehyde (PFA) in phosphate buffered saline (PBS, pH = 7.4). In short, it was placed on its back with the paws restrained. A cut was made at the level of the xyphoid process with small sharp scissors to expose it, followed by lateral cuts to expose the rib cage. The xyphoid process was lifted and cuts were made laterally along the diaphragm and superiorly through the ribs. The anterior rib cage was elevated superiorly and stabilized to expose the chest cavity and heart. A 29-guage needle was carefully placed into the ventricle and a cut was made in the right atrium with rounded scissors. 20 mL of PBS were passed through the needle to flush out the blood, followed by 30 mL of 4% PFA.

The head was cut at the base of the neck using sharp scissors, and the soft tissue and skull top were removed. The brain and skull base were then placed in 4% PFA overnight at 4 °C. To decalcify the skull base and the cochleae, the murine heads were placed in 0.12 M ethylene-diamine-tetra-acetic acid (EDTA) for 14 days at room temperature on a shaker. After dehydration with sucrose 5% (6-12 h, 4 °C) and sucrose 30% (6-12 h, 4 °C), heads were incubated in optimal cutting temperature (O. C. T.) compound (Tissue-Tek) at 4 °C for at least 4 h to allow penetration of the O. C. T into the EAC and skull base structures. The heads were then embedded in O. C. T. at −20 °C and cut into 12-μm sections using a cryostat (Leica Microsystems).

The sections were mounted onto slides and underwent antigen retrieval in 1% citrate buffer at 80 °C for 20 min, then allowed to cool. They were then placed in blocking solution for 1–2 h at room temperature. Slides were then incubated with mouse-anti-Beta-Tubulin III (TuJ, 1:300) (BioLegend) and chicken-anti-GFP (abcam, 1:250) in antibody solution overnight at 4 °C. The slides were then incubated with the appropriate secondary antibodies (Alexa Fluor) at room temperature for 1–2 h, then with DAPI (BD biosciences) 1:1000 for 5 min. Coverslips were applied and left to dry overnight at room temperature before confocal imaging.

Confocal imaging (Leica Microsystems) on coronal sections was performed to analyze cell integration qualitatively by observation of TUJ+/GFP + cells in the cochlear nerve, in addition to their projections towards the modiolous.

## 4 Results

### 4.1 Stereotaxic surgery

A novel stereotaxic approach was developed for minimally invasive targeting of the auditory nerve. This technique enables precise delivery of therapeutic agents without disrupting the delicate cochlear architecture. While the stereotaxic frame provided superior stability, accurate head placement required additional time during initial experiments. Variations in skull dimensions across mouse strains necessitated slight modifications to published coordinates. The coordinates used in the study after adjustments based on our results were AP = −5.07 mm, ML = +2.46 mm, and DV = 5.25 mm ± 0.1 mm (Table 1). Additionally, fixation of the blunt ear bars was critical, as even minor shifts during drilling could alter the injection trajectory. Injection success rate was defined by presence of cells within the auditory nerve trunk at the cochlear aperture. Injection failure included cells found in the wrong location or complete absence of fluorescent signal. Overall, the method demonstrated moderately high reproducibility, with 76.2% of injections accurately targeting the auditory nerve (Table 1).

**Table 1:** Adjusted stereotaxic coordinates and injection success rate.

ML (X) coordinate (mm)	AP (Y) coordinate (mm)	DV (Z) coordinate (mm)
2.46 ± 0.15	−5.07 ± 0.60	5.25 ± 0.39

Total *n* = 21; success rate with mES injections: 76.2% (*n* = 15).

Five animals with no staining observed or staining in the incorrect area observed.

### 4.2 Surgical procedure: transplantation of progenitor cells

**Figure 3:**
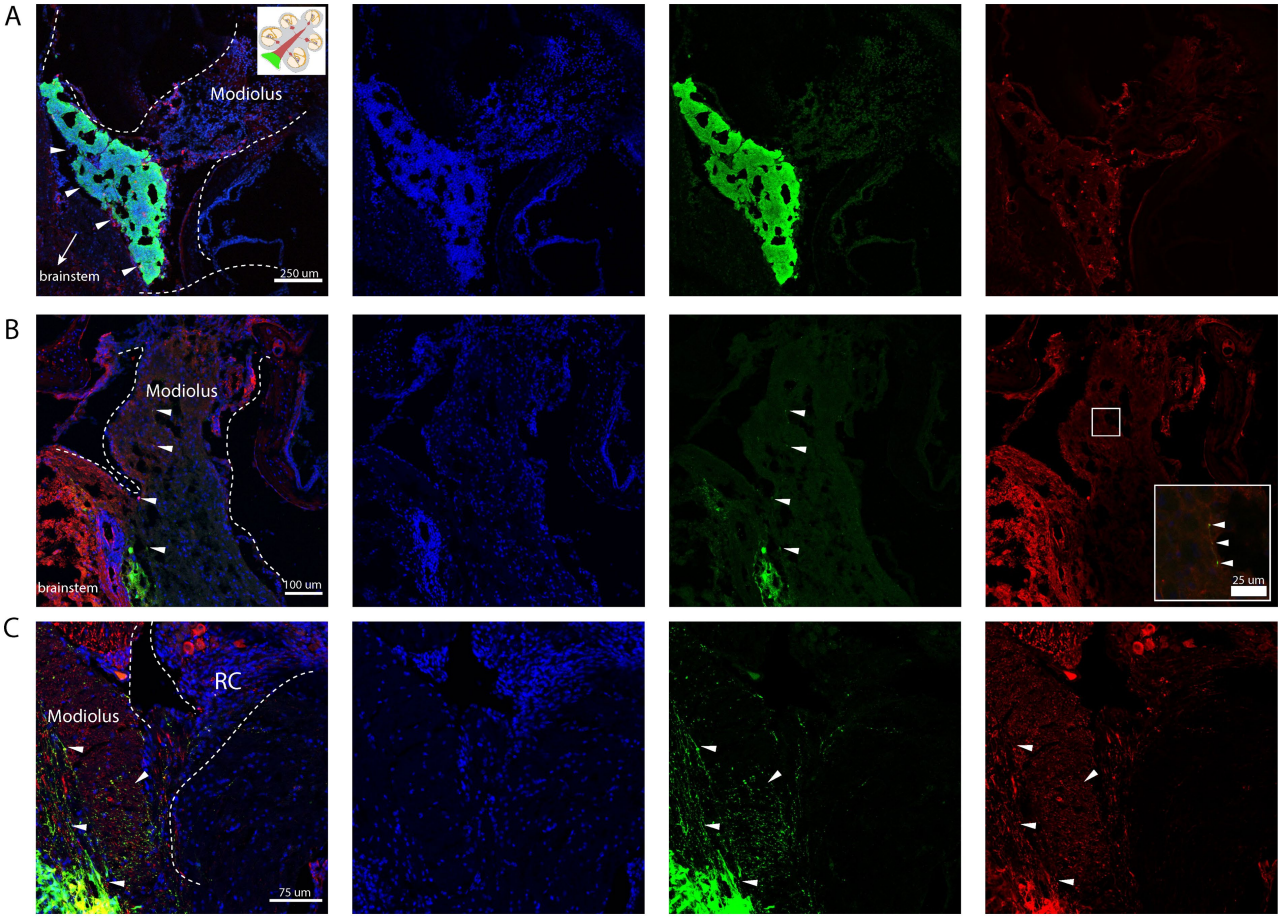
Stereotactic injection of mESC into the proximal auditory nerve. Nuclear counterstain (DAPI, blue), transplanted cells (GFP+, green), Neurons (TUJ+, red). **(A)** Engrafted Tau-EGFP+ progenitor cells in base of the cochlear modiolus (white arrow heads). **(B)** Peripheral projections from the graft extending distally into the modiolus, with few cells exhibiting positive co-staining of TuJ and GFP (white arrow heads). **(C)** Peripheral projections from the graft extending towards Rosenthal’s canal (RC); few surviving neuronal cell bodies can be appreciated in RC (red); few cells exhibiting positive co-staining of TuJ and GFP (white arrow heads). Created in BioRender. Kempfle, J. (2025) https://BioRender.com/4qorwwq.

Immunohistological analysis revealed that transplanted cells successfully engrafted in the cochlear modiolus, particularly near the basal turn (Figure 3A). Tau-EGFP+ cells exhibited neuronal morphology and extended peripheral project ions toward hair cells within the organ of Corti (Figures 3B,C). Co-staining with neuronal markers such as TuJ confirmed differentiation into neural-like cells (Figures 3B,C). Off-target injections were rare, and occurred in the cochlear nucleus, due to injections that were not deep enough in the dorsoventral plane, in addition to the facial nerve in one animal. Cells were frequently seen in Scarpa’s ganglion and the vestibular nerve, in addition to a positive signal in the auditory nerve, which could not be avoided due to the close relationship of the cochlear and vestibular nerves. Adverse effects, observed in 3 mice after surgery, included mild vestibular effects with transient spinning and imbalance. A larger needle size (33-guage) was utilized to prevent shearing of cells. Injection volumes of 1–3 μL were sufficient for cell delivery without causing mass effect or post-op complications.

## 5 Discussion

Stereotaxic injection targeting the auditory nerve represents a transformative advance in the field of inner ear therapeutics, offering a minimally invasive and highly precise delivery system for regenerative therapies of auditory neurons. This study demonstrates the feasibility of delivering stem cell-derived neural progenitor cells directly into auditory nerve trunk at the cochlear aperture using a novel stereotaxic protocol. By circumventing the challenges of traditional surgical approaches to the cochlea, which primarily include indirect delivery of cells and may result in cochlear damage, this method ensures targeted, minimally invasive access while preserving the integrity of surrounding cochlear structures.

The use of predefined coordinates, adapted from the Paxinos and Franklin Mouse Brain Atlas and modified for the CBA/CaJ strain, allowed precise stereotaxic targeting of the auditory nerve trunk. The consistency of successful targeting highlights the reproducibility of this approach. Challenges, such as variations in skull morphology across mouse strains and slight deviations in coordinate depths were minor in our wildtype models, but strain-specific adaptation of this approach may be necessary for certain transgenic lines.

The oblique orientation and small diameter of the auditory nerve relative to other central nervous system (CNS) targets present unique challenges in stereotaxic delivery. Minor inaccuracies in positioning, particularly in the dorsoventral plane, could lead to occasional misplacement into adjacent structures such as the cerebellopontine angle or brainstem. This limitation underscores the critical role of precise head alignment during surgical preparation, requiring consistent leveling of the skull via ear bars and a tooth bar. In skilled hands, however, the technique proved highly effective and reproducible.

Timing is another critical component of experimental design for any regenerative therapy. The cochlear microenvironment undergoes significant changes following injury to sensory cells, creating a window of heightened receptivity to regenerative interventions. Specifically, inner ear glial cells exhibit proliferation and dedifferentiation in the days and weeks following auditory nerve damage, rendering them more susceptible to endogenous regeneration cues and facilitating the integration of transplanted progenitors (46). Therefore, it was imperative for cell transplantation to occur shortly after SGN degeneration but prior to glial scar formation, which we determined to be within 7–10 days after induction of the model of AN by Oubain application to the round window membrane (46).

One of the key advantages of this method lies in its minimally invasive nature, minimizing risks of iatrogenic damage to the cochlea or middle ear. Traditional approaches, such as cochleostomy or round window membrane injection, carry a substantial risk of fluid homeostasis disruption, resulting in secondary damage to hair cells and residual hearing. In contrast, our stereotaxic approach avoids direct manipulation of the cochlear labyrinth, significantly reducing the likelihood of SNHL due to procedural trauma. Postoperative assessments confirmed the preservation of OHC function, as indicated by stable DPOAEs, even in animals undergoing high-precision injections.

While the overall procedure proved safe and effective, certain theoretical risks remain. The proximity of the auditory nerve to vascular structures, including the transverse sinus, necessitates careful management of venous bleeding during craniotomy. Additionally, the use of blunt ear bars mitigates the risk of trauma to the tympanic membrane and middle ear but requires experience for proper placement without compromising fixation. Further refinements in head-holding apparatuses and automated robotic assistance could further enhance procedural safety and accuracy.

This study extends the use of stereotaxic techniques to include successful delivery of agents to the neural component of the inner ear. Transplantation of stem-cell derived neural progenitor cells demonstrated initial integration into the cochlear nerve trunk, with grafted cells extending peripheral projections toward sensory hair cells, and central projections towards the brainstem. This finding is particularly promising for addressing SNHL, as it suggests the potential for functional circuit repair through SGN replacement. This technique could be extended to delivery of gene therapy vectors, small molecules, antisense oligonucleotides, or CRISPR-based gene-editing tools. The ability to target SGNs directly may prove especially valuable in treating ANSD disorders, where current interventions like cochlear implants are less effective due to neuronal damage.

Despite its potential, the stereotaxic method harbors limitations. The auditory nerve’s small size and complex trajectory pose constraints on injection volume and reduces success rate of the procedure. Our findings suggest that three microliters for cells reliably demonstrated a cell deposit at the cochlear aperture and the auditory nerve, without inducing mechanical damage or wide dispersion of cells. However, these parameters may need adjustment for other animal models or transgenic lines. Another limitation lies in the lack of age-specific data. While this study exclusively employed adult mice, developmental or age-related changes in auditory nerve anatomy may require re-evaluation of stereotaxic coordinates. Additionally, further research is required to test the method’s applicability across different transgenic mouse models.

In summary, the stereotaxic injection protocol described here represents a significant advancement in inner ear cellular therapies. By enabling precise and minimally invasive targeting of the auditory nerve, this method opens new avenues for future regenerative research in auditory neuropathy and sensorineural hearing loss.

## Data Availability

The raw data supporting the conclusions of this article will be made available by the authors, without undue reservation.

## Glossary

ABR: Auditory brainstem response
AN: Auditory neuropathy
ANSD: Auditory neuropathy spectrum disorders
AP: Anteroposterior
BDNF: Brain-derived neurotrophic factor
CNS: Central nervous system
dB: Decibel
DPOAE: Distortion product otoacoustic emissions
DV: Dorsoventral
ESC: Embryonic stem cells
HC: Hair cell
i.p.: Intraperitoneal
mESC: Mouse embryonic stem cells
ML: Mediolateral
NT-3: Neurotrophin-3
OHC: Outer hair cell
PBS: Phosphate buffered saline
PFA: Paraformaldehyde
SGN: Spiral ganglion neuron
SNHL: Sensorineural hearing loss
SPL: sound pressure level
